# Dysregulation of alternative splicing is associated with the pathogenesis of ulcerative colitis

**DOI:** 10.1186/s12938-021-00959-4

**Published:** 2021-11-27

**Authors:** Daowei Li, Yue Tan

**Affiliations:** 1 Department of Radiology, The People’s Hospital of China Medical University & The People’s Hospital of Liaoning Province, No. 33, Wenyi Road, Shenhe District, Shenyang, 110016 China; 2 Department of Gastroenterology, Shengjing Hospital of China Medical University, Tiexi District, 39 Huaxiang Road, Shenyang, 110022 China

**Keywords:** Ulcerative colitis, Alternative splicing, Posttranscriptional regulation, RNA-Seq

## Abstract

**Background:**

Although numerous risk loci for ulcerative colitis (UC) have been identified in the human genome, the pathogenesis of UC remains unclear. Recently, multiple transcriptomic analyses have shown that aberrant gene expression in the colon tissues of UC patients is associated with disease progression. A pioneering study also demonstrated that altered post-transcriptional regulation is involved in the progression of UC. Here, we provide a genome-wide analysis of alternative splicing (AS) signatures in UC patients. We analyzed three datasets containing 74 tissue samples from UC patients and identified over 2000 significant AS events.

**Results:**

Skipped exon and alternative first exon were the two most significantly altered AS events in UC patients. The immune response-related pathways were remarkably enriched in the UC-related AS events. Genes with significant AS events were more likely to be dysregulated at the expression level.

**Conclusions:**

We present a genomic landscape of AS events in UC patients based on a combined analysis of two cohorts. Our results indicate that dysregulation of AS may have a pivotal role in determining the pathogenesis of UC. In addition, our study uncovers genes with potential therapeutic implications for UC treatment.

**Supplementary Information:**

The online version contains supplementary material available at 10.1186/s12938-021-00959-4.

## Background

Ulcerative colitis (UC), a subtype of inflammatory bowel disease (IBD), has become a global disease [1]. The pathogenesis of UC is complicated and involves shifts in interactions among intestinal microbes, the host’s genetic background, and environmental cues, resulting in the chronic activation of the mucosal immune system [2, 3]. Although multiple genome-wide association studies have identified hundreds of associated genetic risk loci in patients with UC [4], its pathogenesis remains unclear.

Recently, several transcriptomic studies have been performed to determine gene expression alterations in patients with different subtypes of IBD. Planell et al. analyzed colonic biopsies from patients with active or inactive UC and healthy controls using microarrays. They discovered that several genes were deregulated in the inactive UC despite histological recovery [5]. Another microarray study demonstrated that expression of INF‑γ and IL‑17 was comparably elevated in both inflamed and unaffected colon mucosa from patients with IBD, indicating that the inflammatory response is not limited to the endoscopic lesions [6]. Smith et al. identified that dysregulation of BMP/retinoic acid inducible neural specific 3 plays a role in the pathogenesis of UC [7]. A more recent study that analyzed the colonic mucosal transcriptome of patients with long-duration UC also identified dysregulated gene expression and pathways in long-duration UC patients compared with the short-duration patients [8]. Overall, these studies suggest that transcriptional regulation has a crucial role in determining the etiology of UC.

Alternative splicing (AS) of the precursor mRNA is one of the essential mechanisms for increasing protein diversity and regulating the intricate protein-RNA interaction network [9, 10]. Almost 95% of all human genes with multiple exons are involved in AS events [11]. Increasing evidence implies that AS plays crucial roles in many biological events, such as oncogenic processes, including cell proliferation, cell apoptosis, hypoxia, immune escape, and metastasis [12, 13]. It also has an essential role in fundamental developmental processes and tissue identity [14]. A recent pioneering study, which profiled the AS events in IBDs, showed that 47 splicing factors and 33 intron retention events were dysregulated in the mucosal tissue of patients with IBD [15]. Another array-based pioneer study found more than 392 differentially expressed genes between long- and short-duration UC patients. Among them, most of them were associated with a dysregulated AS network [8]. However, the former study did not use a next-generation sequencing approach, and the latter mainly focused on comparing long-duration UC and short-duration UC.

To elucidate how AS participates in the pathogenesis of UC, we first obtained public mRNA-Seq data from the NCBI GEO dataset GSE137344, which included 44 mRNA expression data from UC patients and 37 from the controls. Using Miso and related AS event analysis software [16], we identified 8 AS types and 2,385 significant AS events in UC patients versus the control. For these AS events, 110 biological pathways were significantly enriched in the UC patients, of which some were highly involved in chronic inflammation/immune response pathways. To validate the AS events identified in the patients, we performed an mRNA-Seq experiment on colon tissues from our cohort of UC patients and a healthy control group (4 patients vs. 4 healthy individuals). Fifty-seven percent of the genes that involved significant AS events in our experiment also had significant AS events in the GSE137344 dataset. Immune response-related pathways were also the most significantly enriched terms in our dataset. To further demonstrate the potential role of AS in UC progression, we also performed a validation experiment comparing the AS of the two clusters of UC patients with different disease progressions. Overall, we provide a comprehensive analysis of AS events in patients with UC by comparing multiple datasets. We believe our results shed light on the mechanism of post-transcriptional regulation in the pathogenesis of UC.

## Results

### Types of alternative splicing in the GSE137344 public dataset

**Fig. 1:**
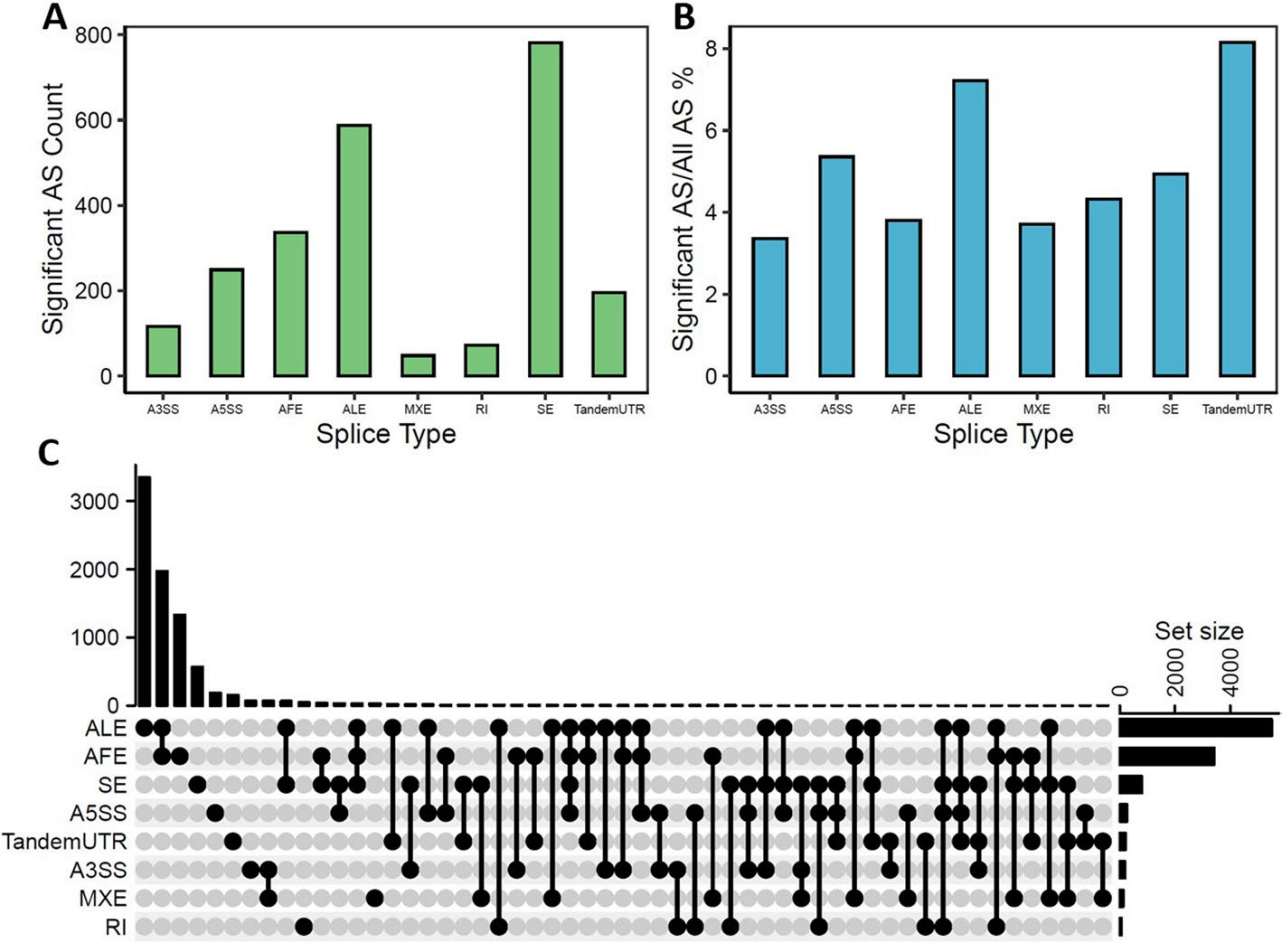
Alternative splicing identification in Ulcerative Colitis disease samples and normal samples. **A** Significant AS event counts in different AS types. **B** The ratio of related AS events over all AS events in different AS types. **C** Distribution of AS types and intersection between gene symbols of 2385 related AS events

Eight types of AS events were discovered in UC samples from the GSE137344 public dataset: alternative 3’splice site, alternative 5’splice site, alternative first exon (AFE), alternative last exon (ALE), mutually exclusive exons (MXE), retrained introns (RI), skipped exon (SE), and tandem 3′ UTR (Fig. 1A). Of these, 2358 AS events were significant (Additional file 2: Table S1) (Additional file 1: Fig. S1) compared with the control samples. Most belonged to the SE type, but the total number of SE events was relatively low versus other events (Fig. 1B). Conversely, only 8.1% of tandem 3′ UTR events and 7.2% of ALE events were significant, yet these AS events overnumbered others. The distribution and intersection of gene symbols between the different event types for related AS events are shown in Fig. 1C. Since most ALE and AFE AS events were identified across multiple genes, they were mapped to over 5,000 and 3,000 genes, respectively, and hence had the most intersections.

### Pathway analysis of the GSE137344 public dataset

**Fig. 2:**
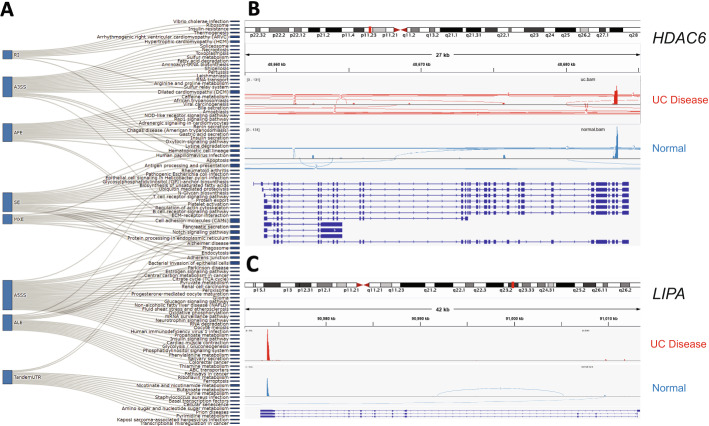
Pathway Analysis. **A** The biological pathways associated with the related AS events in different splice type. **B** HDAC6 mRNA expression in UC samples and normal samples. **C** LIPA mRNA expression in UC samples and normal samples

The AS events related to UC were enriched with 110 different biological pathways (Additional file 3: Table S2), and these were mainly associated with only one splicing type. By contrast, “antigen processing and presentation” and “ribosomes” were the only pathways affected by three splicing types (Fig. 2A). “Systemic lupus erythematosus” was the most significantly enriched term, with a *P* value less than 5 × 10^−20^. Interestingly, a recent study showed that patients with SLE had a greater prevalence of IBD than the controls [17]. The second most enriched term was “Alcoholism,” with a *P* value less than 3 × 10^−6^. Next, we combined the results of AS event pathway analysis with the RNA-Seq expression results. We identified two inflammation-related genes containing UC-related AS events in these pathways. The first, encoding histone deacetylase 6 (*HDAC6*), had an ALE event, and the second, encoding a lipase A (*LIPA*), had a tandem 3′ UTR. A previous study showed that *HDAC6* is involved in the alcoholism pathway and thus which was associated with chronic inflammation [18]. LIPA was previously associated with steroid biosynthesis which was also considered to be involved in modulating inflammation [19]. Two genes in different sample groups showed significant differential expression on RNA level (Fig. 2B, C).

### Splicing types in the 8-sample mRNA-seq experiment

**Fig. 3:**
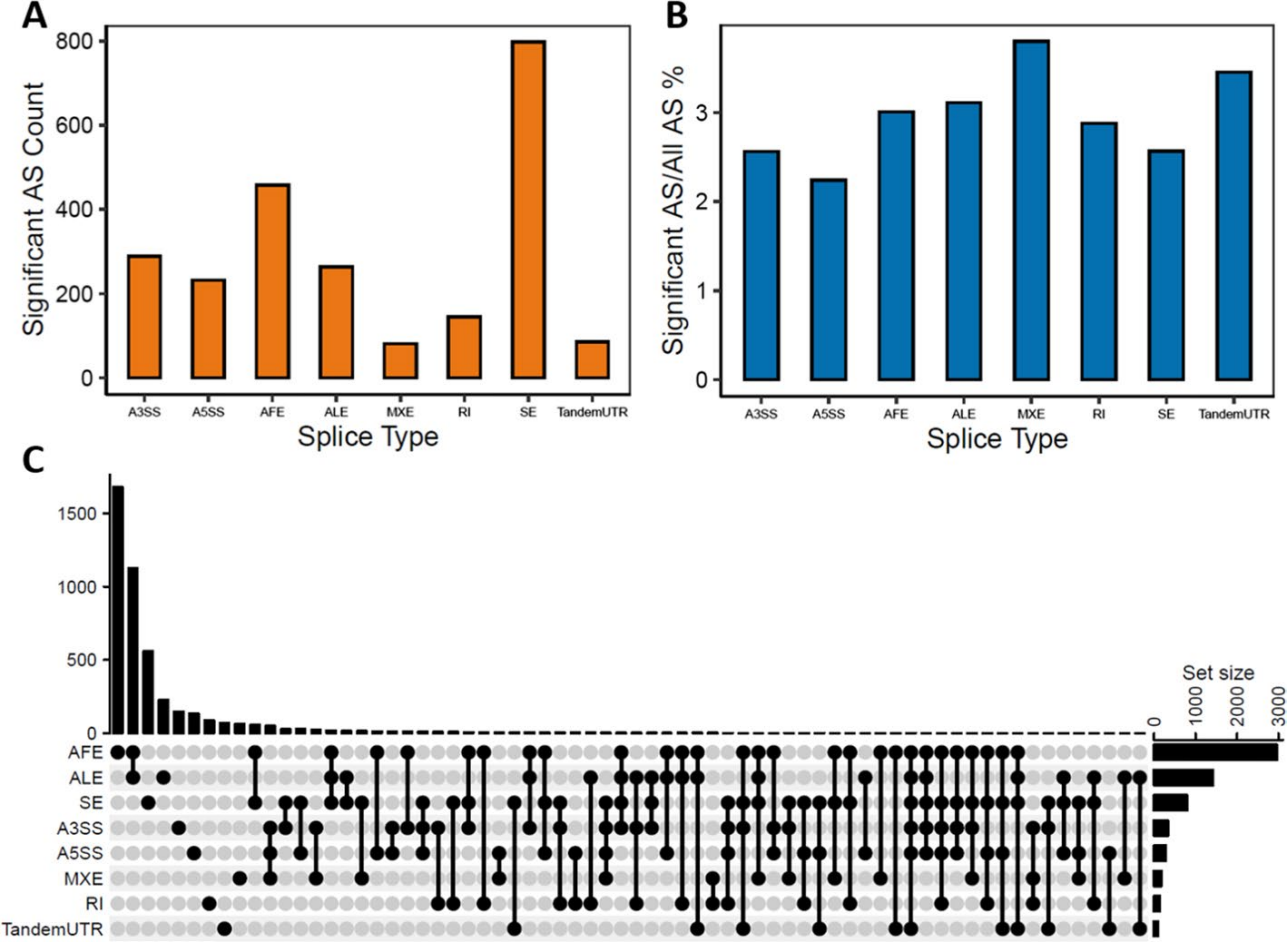
Alternative splicing identification in Ulcerative Colitis disease samples and normal samples in validation experiments. **A** Significant AS event counts in different AS types. **B** The ratio of related AS events over all AS events in different AS types. **C** Distribution of AS types and intersection between gene symbols of 2352 related AS events

To validate AS events that indeed exists comprehensively in the UC, we performed another RNA-seq experiment on four UC patients and four normal samples from Shengjing Hospital of China Medical University. We analyzed the data of our experiment using the same steps as for the GSE137344 dataset and discovered a set of 2352 significant AS events (Additional file 4: Table S3) (Additional file 1: Fig. S2). As in the GSE137344 dataset, SE and AFE were the most common AS types, while MXE was the least common (Fig. 3A). However, the ratio of all event types was relatively different compared with the results from the public dataset (Fig. 3B). AFE and ALE were identified across multiple genes, which exhibited the highest number of intersections (Fig. 3C). Interestingly, 57% of the genes with significant AS events in our dataset also had them in the public (Additional file 1: Fig. S3), although they were from different tissues. This result indicates that AS regulation could be more related to disease progression than tissue type.

### Combined analysis of expression and splicing in the 8-sample mRNA-seq dataset

**Fig. 4:**
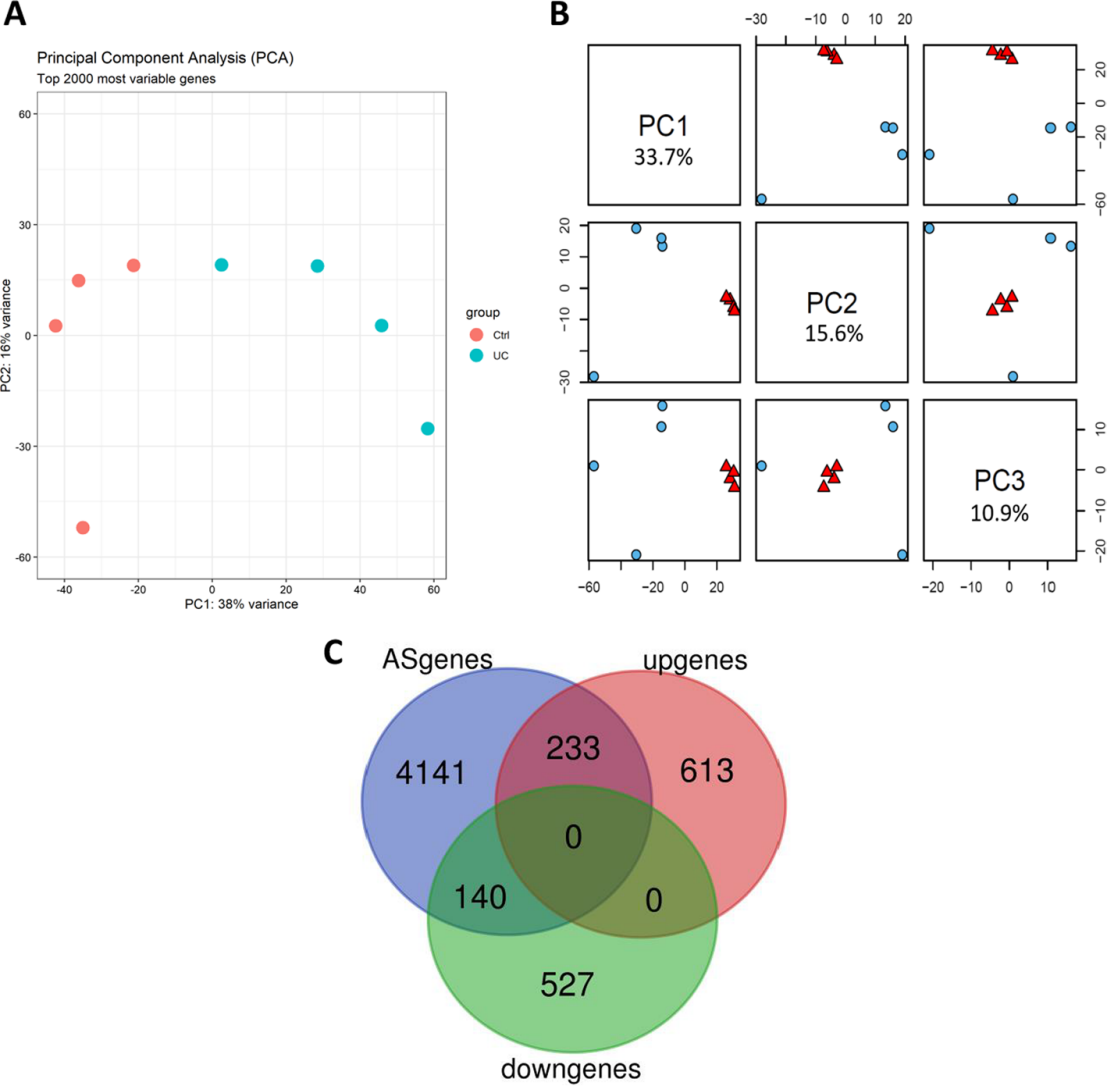
Combined analysis of AS events and expression profile in UC patients. **A** PCA plot of 4 UC versus 4 control expression profile using the top 2000 regulated genes as variables. **B** Scatterplot pairs of first three principal components for Percent Spliced In (PSI) values across 8 samples. The red triangles represent UC disease patients and the blue circle represent normal samples. **C** Venn diagram showed overlapped genes involved in expression regulation and AS regulation

mRNA-Seq experiment on the eight samples identified over 1500 differentially expressed genes (*P* < 0.05; log2 fold change > 0.5) in UC patients compared with controls (Additional file 1: Figure S4). Principal component analysis (PCA) was performed on the expression data of top 2000 genes (Fig. 4A). We also performed a PCA analysis of the AS events to characterize these events between UC and control samples. We summarized 1731 related AS events across all eight samples with Percent Spliced In (PSI) values generated by MISO software. PC1 and PC3 accounted for 60% of the variance (Fig. 4B), and PC1, PC2, and PC3 captured the biological difference between UC patients and control. These results thus indicate that AS patterns and expression profiles demonstrate the biological differences between the UC samples and controls.

We next generated a Venn diagram to examine whether the expression levels of the genes with significant AS events also showed significant changes in expression. It showed that 140 of 667 downregulated genes and 233 of 846 upregulated genes also had AS events in UC patients. This result indicates that at least some of the changes in gene expression are due to dysregulation of AS (Fig. 4C).

### Biological pathway analysis

**Fig. 5:**
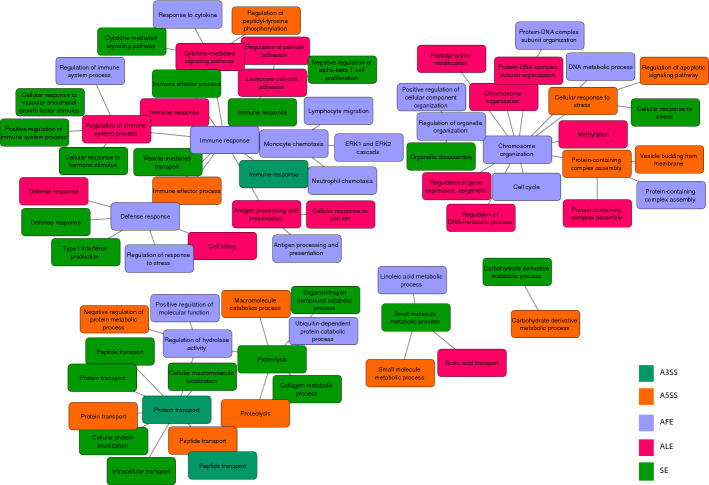
Biological process network enrichment result from Gene Ontology analysis

We performed a biological pathway analysis of the gene list with 2352 significant AS events. Since only less than 200 related genes had MXE, RI and tandem 3′ UTR event types, we failed to identify any significantly enriched biological process among these genes. Among the other five AS type events, multiple pathways were highly enriched in terms of biological process (Fig. 5). Among those pathways, “immune response” was enriched most significantly. Besides, LIPA, which was identified to be differentially expressed as well as had significant AS events in the public dataset, was also identified to involve a tandem 3’UTR event and showed the significant differential expression on RNA level in the validation dataset (Additional file 5: Table S4) (Additional file 1: Fig. S5). These results suggested that the dysregulated AS, which was similar to the expression level itself, was strongly associated with altered immune response in UC patients.

**Fig. 6:**
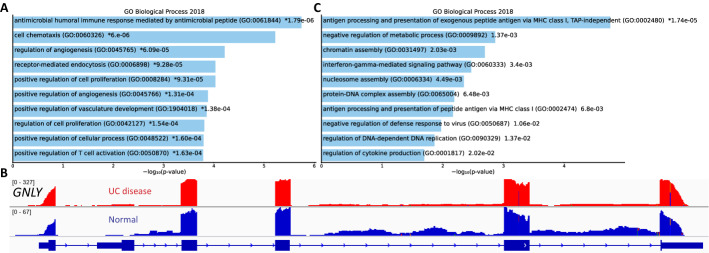
Analysis of GO enrichment in multiple experiments. **A** Top 10 enriched GO Biological Process terms of related genes of 201 uniquely AS events in 4 samples of UC patients. **B** Visualization of AS events on GNLY gene. **C** Top enriched GO terms of 111 significantly expressed AS events between two clusters of UC patients with different disease severity

We next performed gene ontologies (GO) analysis on the 8-sample dataset based on the enrichment of 201 unique AS events that were only discovered in the normal group or in the UC group. The top 10 enriched GO biological process terms (Fig. 6A, Additional file 6: Table S5) reflect the immune system response and cell chemotaxis in the UC patients. GNLY is one of the genes that has unique AS events in term GO:0061844. The product of this gene is a member of the saposin-like protein (SAPLIP) family. It is an antimicrobial protein that present in the granules of human cytotoxic T lymphocytes, as well as in the natural killer (NK) cells, that can also activate antigen-presenting cells through TLR4 [20]. In Fig. 6B, we presented different AS events of this gene between the normal and UC patients as captured in the read coverage track (Fig. 6B). Two 3′ intron retention events were identified only in healthy tissues. Recent studies have shown that intron retention may affect transcriptional efficiency [21]. Therefore, we speculated that this unique AS event in healthy individuals limits *GNLY* transcription, while its absence in UC patients increases *GNLY* transcription. We also compared the AS events in two clusters of UC patients with different disease progression statuses from the GSE130038 study and identified 111 significant AS events between the two clusters. Pathway enrichment analysis also identified specific GO terms related to the disease progression (Fig. 6C). These results suggest that AS may play a vital role in the pathogenesis of UC, acting as an indicator of disease progression.

## Discussion

Dysregulation of AS is strongly associated with pathogenesis of many human diseases. Emerging evidence shows that targeting cellular mRNA splicing may lead to novel therapeutics [22]. Multiple studies have demonstrated the roles of dysregulated AS in many human diseases, including cancer, immune and infectious diseases, and neurodegenerative disorders [23–25]. Indeed, two pioneering studies [8, 15] have identified that dysregulation of AS may be associated with the pathogenesis of IBD. However, neither study performed enrichment analysis of the AS-related genes nor compared disease and control samples using non-array-based methods. One of the studies only focused on intron retention events, while ours analyzed all main types of AS events. In addition, by analyzing two different datasets, we revealed that many AS-related genes overlapped between the two cohorts, indicating that dysregulation of AS is strongly associated with the pathogenesis of UC.

We also discovered that SE and AFE were the two most significantly enriched AS events in both datasets; thus, the transcriptome of UC patients may be more complex and chaotic than that of healthy individuals. Both SE and AFE yield aberrant mRNA isoforms, which may activate a negative feedback regulation of transcription, for instance, degradation [26]. Many genes that showed significant AS events also had significant changes in expression levels. Although it is uncertain whether these changes are due to dysregulated AS events, we can conclude a significant positive association between them. Among the 23,519 genes detected in our mRNA-Seq dataset, 1513 were significantly dysregulated *(P* < 0.05). Of the 4514 genes with AS events, 373 had significantly dysregulated expression; therefore, genes with AS events are likely to have altered gene expression (Fisher’s exact test *P* value = 0.0001).

Among the AS-related significantly enriched pathways in our dataset, immune response and related pathways had the largest network and most significantly enriched terms. Of the genes identified in the enriched pathways, we were particularly interested in two, *HDAC6* and *LIPA*, as both had significant AS events (tandem 3′ UTR and ALE) and were also involved in inflammation and immune response. HDAC6 is a member of the histone deacetylase family proposed as anti-inflammatory therapy for treating colon inflammation that may progress to IBD [27, 28]. LIPA is a lysosomal acid lipase that catalyzes the hydrolysis of triglycerides and cholesteryl esters. A recent study demonstrated that lysosomal cholesterol hydrolysis is critical for preventing metabolic inflammation. LIPA functions as a key regulator of the process, as its inhibition causes defective clearance of apoptotic lymphocytes [29]. In the two datasets, both the expression level and AS pattern of LIPA were significantly dysregulated. Overall, our results suggest that targeting the dysregulated AS of HDAC6 or LIPA is a potential strategy for developing novel therapies.

Although we presented a comprehensive AS analysis of the ileum and colon tissues of patients with UC, our study had certain limitations. For example, we did not experimentally validate the AS events detected in our experiments nor whether these events affect protein diversity. Moreover, we propose a RIP-Seq or CLIP-Seq study to uncover the potential splicing factors involved in the underlying mechanism of AS alteration in UC patients.

## Conclusion

This study presents a genomic landscape of AS events in UC patients based on a combined analysis of two cohorts. Our data suggest a strong association between dysregulation of AS and the pathogenesis of UC. Dysregulated AS of HDAC6 or LIPA may be potential targets for developing novel therapies. Future drug screening studies should focus on AS-related genes and AS-related splicing factors.

## Materials and methods

### Sample information and collection

Four patients with UC from Shengjing Hospital of China Medical University were recruited between June and September 2020 and served as the experimental group. All the patients had an established diagnosis of UC based on endoscopic and histological assessments. Colonic biopsy specimens were taken from the rectum, ulcer margin of the sigmoid colon, and inflamed portions.

Four patients with normal distal colon confirmed by surgical pathology served as the control group.

The study was approved by the institutional review board of Shengjing Hospital of China Medical University, and informed consent was obtained from each patient.

### Public dataset preparation

We obtained RNA-seq data from NCBI (GSE137344). This dataset included 44 mRNA expression data of of ileum tissues from patients and 37 from healthy individuals (we chose this dataset because the mRNA expression is restricted to the gastrointestinal tract and extends to the terminal ileum [30, 31]). Fastq files were aligned with the human Hg19 genome using STAR-2.7.1a. The indexed.bam files were generated using Samtools (1.10). Using the above pipeline, we also analyzed the GSE130038 dataset [32] that contained RNA-Seq data of colon tissues.

### Identification of critical AS events

Using an in-house MISO pipeline, 8 AS types and 60,690 AS events were identified with the MISO (0.5.4) package [16] for 81 mRNA samples from the GSE137344 RNA dataset. The level of AS events was defined as the percentage spliced in (PSI). To obtain differentially expressed AS events between UC patients and controls, we applied the Wilcoxon test on all AS events with at least three patients and one control sample. Significant events were defined as those with *P* < 0.05. Among them, 2385 AS events had *P* values less than 0.05 between 44 UC samples and 37 control samples and thus were defined as related or significant AS events. We removed those AS events that occurred in less than three patients and one control sample.

The above approach was also applied to our RNA-Seq dataset, in which 8 AS types and 94,815 AS events were identified for eight samples. A total of 2352 AS events were significant, with *P* < 0.05 between four UC samples and four controls. Those AS events that occurred in less than two patients and one control sample were excluded. Optimization algorithm [33, 34] and feature selection related algorithm [35] were referenced to enhance our analysis performance.

### Pathway analysis

Significant AS-event-related genes were chosen in pathway analysis. These are included in the Additional file 8: Table S7, Additional file 9: Table S8. Pathway analysis of the GSE137344 dataset was performed using Enrichr with the Kyoto Encyclopedia of Genes and Genomes (KEGG) pathway library. Only pathways with a *P* value less than 0.05 were considered related. For our dataset, the pathway analysis was done with WebGestalt (2019) and SUMMER tools. We selected overrepresentation analysis (ORA) as the enrichment method and gene ontology biological process as the functional database in the WebGestalt analysis. The enriched categories with a gene size less than 5 and a false discovery rate (FDR) above 0.05 were excluded from the WebGestalt results. The SUMMER analysis results were input into Cytoscape (3.8.0) software to modify the color and text size of the network.

### Gene ontology analysis

Gene ontology analysis of our dataset was performed using Enrichr tool, and the bar plot of the top 10 enriched terms was created using Enrichr Appyter.

### RNA-Seq analysis pipeline

The RNA-Seq library was prepared using the NEBNext Ultra RNA with Poly-A selection kit and sequenced on an Illumina HiSeq 4000 platform (Genergy, Shanghai). Kallisto [36] software was used to quantify RNA-Seq counts. Differential gene expression was determined with log2 fold change > 1.5 and *P* < 0.05, for genes with > 1 count per million. After Benjamini–Hochberg correction for multi-testing, any gene with a *P* value greater than the false discovery rate (FDR) was deemed significantly differentially expressed under the test conditions as compared with the controls.

### Visualization

Plotly was used to generate a Sankey diagram. UpSet plots were generated using the ComplexHeatmap [37] in RStudio (1.2.5042). To avoid random error, we randomly selected and merged RNA-Seq alignment data of three UC samples and three control samples from the GSE137344 RNA dataset to generate Sashimi plots by IGV (version 2.8.3). All other graphs were plotted using RStudio (1.2.5042). Venn diagrams were plotted using online software (http://bioinformatics.psb.ugent.be/webtools/Venn/).

## Supplementary Information

**Additional file 1: Figure S1.** PSI comparison of significant AS event between UC and control samples in the GSE137344 public dataset. **Figure S2.** PSI comparison of significant AS event between UC and control samples in the 8-sample mRNA-seq experiment. **Figure S3.** Genes with significant AS event that overlap with the public dataset. **Figure S4.** Volcano plot showed more than 1,654 genes were significantly regulated in UC patients compared to the control. **Figure S5.** Sashimi plot presented tandem 3’UTR events in LIPA gene in our validation experiments.

**Additional file 2: Table S1.** 2385 significant AS events from GSE137344.

**Additional file 3: Table S2.** Pathways which are significantly regulated via Enrichr analysis (GSE137344).

**Additional file 4: Table S3.** Validation experiments results of 2352 related AS events.

**Additional file 5: Table S4.** Raw counts of 4 significantly dysregulated splicing factors expression data from the public dataset and the validation experiment RNA-seq (UC versus Control); FC = Foldchange. The splicing events on mRNA are mainly regulated by splicing factors. We collected 404 splicing factors from (PMID: 29617667).

**Additional file 6: Table S5.** Pathways which are uniquely regulated in validation 4 UC patients via Enrichr analysis

**Additional file 7: Table S6.** Pathways which are significantly regulated via Enrichr analysis (Validation cohort).

**Additional file 8: Table S7.** Gene list of significant AS events for KEGG and GO analysis (GSE137344).

**Additional file 9: Table S8.** Gene list of significant AS events for KEGG and GO analysis (validation cohort).

## Data Availability

Data for 4 UC validation sequencing are available from the corresponding author on request. Please contact author for data requests.

## Abbreviations

UC: Ulcerative colitis
AS: Alternative splicing
IBD: Inflammatory bowel disease
AFE: Alternative first exon
SE: Skipped exon
RI: Retrained introns
MXE: Mutually exclusive exons
ALE: Alternative last exon
NK cell: Natural killer cell

## References

[1] M'Koma AE (2013). Inflammatory bowel disease: an expanding global health problem. Clin Med Insights Gastroenterol.

[2] Jostins L, Ripke S, Weersma RK, Duerr RH, McGovern DP, Hui KY, Lee JC, Schumm LP, Sharma Y, Anderson CA (2012). Host-microbe interactions have shaped the genetic architecture of inflammatory bowel disease. Nature.

[3] Morgan XC, Tickle TL, Sokol H, Gevers D, Devaney KL, Ward DV, Reyes JA, Shah SA, LeLeiko N, Snapper SB (2012). Dysfunction of the intestinal microbiome in inflammatory bowel disease and treatment. Genome Biol.

[4] de Lange KM, Moutsianas L, Lee JC, Lamb CA, Luo Y, Kennedy NA, Jostins L, Rice DL, Gutierrez-Achury J, Ji SG (2017). Genome-wide association study implicates immune activation of multiple integrin genes in inflammatory bowel disease. Nat Genet.

[5] Planell N, Lozano JJ, Mora-Buch R, Masamunt MC, Jimeno M, Ordás I, Esteller M, Ricart E, Piqué JM, Panés J, Salas A (2013). Transcriptional analysis of the intestinal mucosa of patients with ulcerative colitis in remission reveals lasting epithelial cell alterations. Gut.

[6] Xu L, Ma L, Lian J, Yang J, Chen S (2016). Gene expression alterations in inflamed and unaffected colon mucosa from patients with mild inflammatory bowel disease. Mol Med Rep.

[7] Smith PJ, Levine AP, Dunne J, Guilhamon P, Turmaine M, Sewell GW, O’Shea NR, Vega R, Paterson JC, Oukrif D (2014). Mucosal transcriptomics implicates under expression of BRINP3 in the pathogenesis of ulcerative colitis. Inflamm Bowel Dis..

[8] Low END, Mokhtar NM, Wong Z, Raja Ali RA (2019). Colonic mucosal transcriptomic changes in patients with long-duration ulcerative colitis revealed colitis-associated cancer pathways. J Crohns Colitis.

[9] Wang Y, Liu J, Huang B, Xu YM, Li J, Huang LF, Lin J, Zhang J, Min QH, Yang WM (2015). Mechanism of alternative splicing and its regulation. Biomedical reports.

[10] Chen M, Manley JL (2009). Mechanisms of alternative splicing regulation: insights from molecular and genomics approaches. Nat Rev Mol Cell Biol.

[11] Black DL (2003). Mechanisms of alternative pre-messenger RNA splicing. Annu Rev Biochem.

[12] David CJ, Manley JL (2010). Alternative pre-mRNA splicing regulation in cancer: pathways and programs unhinged. Genes Dev.

[13] Oltean S, Bates DO (2014). Hallmarks of alternative splicing in cancer. Oncogene.

[14] Baralle FE, Giudice J (2017). Alternative splicing as a regulator of development and tissue identity. Nat Rev Mol Cell Biol.

[15] Häsler R, Kerick M, Mah N, Hultschig C, Richter G, Bretz F, Sina C, Lehrach H, Nietfeld W, Schreiber S, Rosenstiel P (2011). Alterations of pre-mRNA splicing in human inflammatory bowel disease. Eur J Cell Biol.

[16] Katz Y, Wang ET, Airoldi EM, Burge CB (2010). Analysis and design of RNA sequencing experiments for identifying isoform regulation. Nat Methods.

[17] Shor DB, Dahan S, Comaneshter D, Cohen AD, Amital H (2016). Does inflammatory bowel disease coexist with systemic lupus erythematosus?. Autoimmun Rev.

[18] Kelley KW, Dantzer R (2011). Alcoholism and inflammation: neuroimmunology of behavioral and mood disorders. Brain Behav Immun.

[19] Hu VW, Nguyen A, Kim KS, Steinberg ME, Sarachana T, Scully MA, Soldin SJ, Luu T, Lee NH (2009). Gene expression profiling of lymphoblasts from autistic and nonaffected sib pairs: altered pathways in neuronal development and steroid biosynthesis. PLoS ONE.

[20] Tewary P, Yang D, de la Rosa G, Li Y, Finn MW, Krensky AM, Clayberger C, Oppenheim JJ (2010). Granulysin activates antigen-presenting cells through TLR4 and acts as an immune alarmin. Blood.

[21] Ni T, Yang W, Han M, Zhang Y, Shen T, Nie H, Zhou Z, Dai Y, Yang Y, Liu P (2016). Global intron retention mediated gene regulation during CD4+ T cell activation. Nucleic Acids Res.

[22] Zhao S (2019). Alternative splicing, RNA-seq and drug discovery. Drug Discovery Today.

[23] Wang G-S, Cooper TA (2007). Splicing in disease: disruption of the splicing code and the decoding machinery. Nat Rev Genet.

[24] Scotti MM, Swanson MS (2016). RNA mis-splicing in disease. Nat Rev Genet.

[25] Kim HK, Pham MHC, Ko KS, Rhee BD, Han J (2018). Alternative splicing isoforms in health and disease. Pflügers Archiv-Eur Jo Physiol.

[26] Bitton DA, Atkinson SR, Rallis C, Smith GC, Ellis DA, Chen YY, Malecki M, Codlin S, Lemay JF, Cotobal C (2015). Widespread exon skipping triggers degradation by nuclear RNA surveillance in fission yeast. Genome Res.

[27] Do A, Reid RC, Lohman RJ, Sweet MJ, Fairlie DP, Iyer A (2017). An HDAC6 inhibitor confers protection and selectively inhibits B-cell infiltration in DSS-induced colitis in mice. J Pharmacol Exp Ther.

[28] Liu T, Wang R, Xu H, Song Y, Qi Y (2017). A Highly potent and selective histone deacetylase 6 inhibitor prevents DSS-induced colitis in mice. Biol Pharm Bull.

[29] Viaud M, Ivanov S, Vujic N, Duta-Mare M, Aira LE, Barouillet T, Garcia E, Orange F, Dugail I, Hainault I (2018). Lysosomal cholesterol hydrolysis couples efferocytosis to anti-inflammatory oxysterol production. Circ Res.

[30] Abdelrazeq AS, Wilson TR, Leitch DL, Lund JN, Leveson SH (2005). Ileitis in ulcerative colitis: is it a backwash?. Dis Colon Rectum.

[31] Haskell H, Andrews CW, Reddy SI, Dendrinos K, Farraye FA, Stucchi AF, Becker JM, Odze RD (2005). Pathologic features and clinical significance of "backwash" ileitis in ulcerative colitis. Am J Surg Pathol.

[32] Eshelman MA, Jeganathan NA, Schieffer KM, Kline BP, Mendenhall M, Deiling S, Harris L, Koltun WA, Yochum GS (2019). Elevated colonic mucin expression correlates with extended time to surgery for ulcerative colitis patients. J Gastrointestin Liver Dis.

[33] Abualigah L, Yousri D, Abd Elaziz M, Ewees AA, Al-qaness MA, Gandomi AH (2021). Aquila Optimizer: a novel meta-heuristic optimization Algorithm. Computers & Industrial Engineering.

[34] Abualigah L, Diabat A, Mirjalili S, Abd Elaziz M, Gandomi AH (2021). The arithmetic optimization algorithm. Comput Methods Appl Mech Eng.

[35] Abualigah LMQ (2019). Feature selection and enhanced krill herd algorithm for text document clustering.

[36] Bray NL, Pimentel H, Melsted P, Pachter L (2016). Near-optimal probabilistic RNA-seq quantification. Nat Biotechnol.

[37] Gu Z, Eils R, Schlesner M (2016). Complex heatmaps reveal patterns and correlations in multidimensional genomic data. Bioinformatics.

