# Supplementary figures and images for: Dysregulation of alternative splicing is associated with the pathogenesis of ulcerative colitis
Source: Biomed Eng Online. 2021 Nov 27;20:121. doi: 10.1186/s12938-021-00959-4 (PMC8627048; doi:10.1186/s12938-021-00959-4)

S1

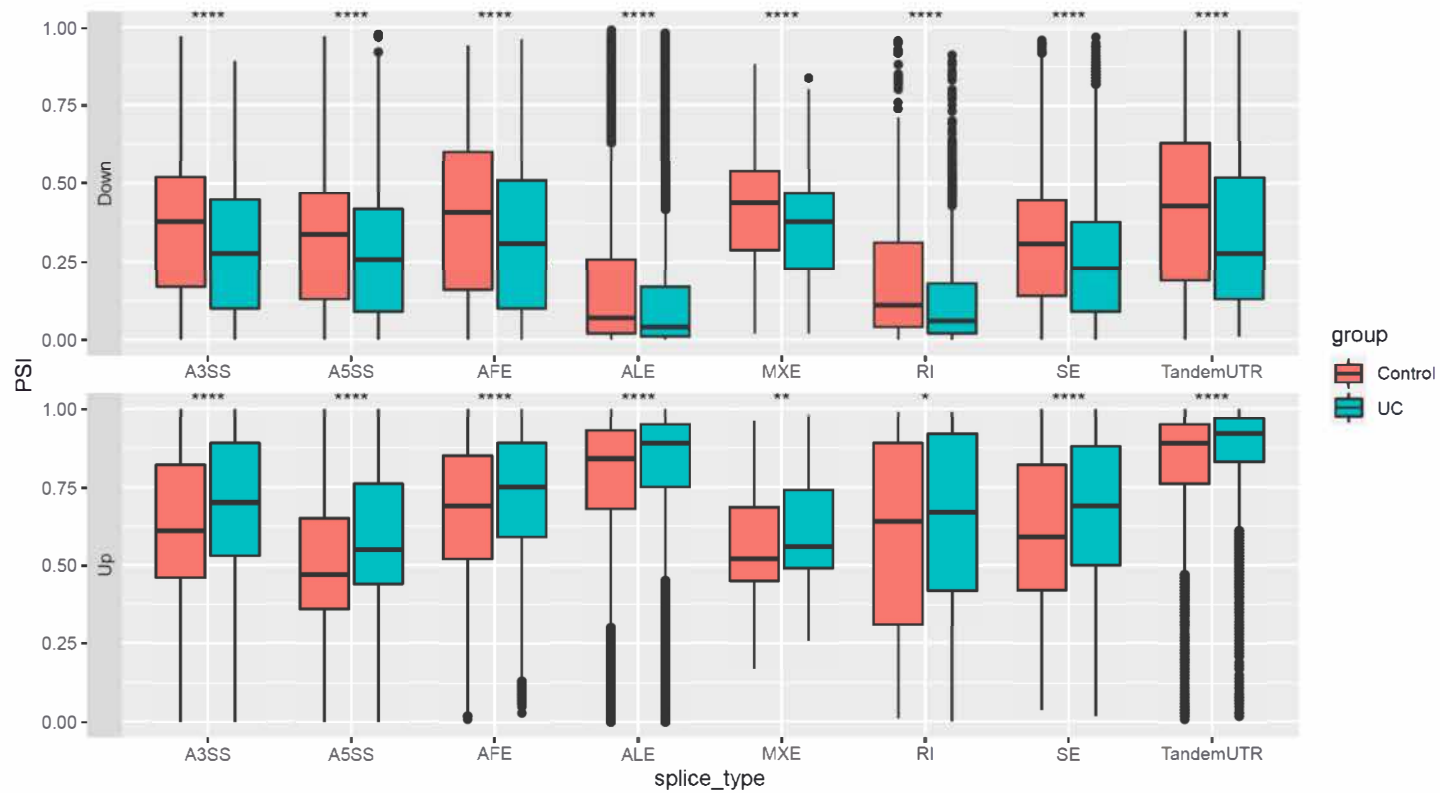

S2

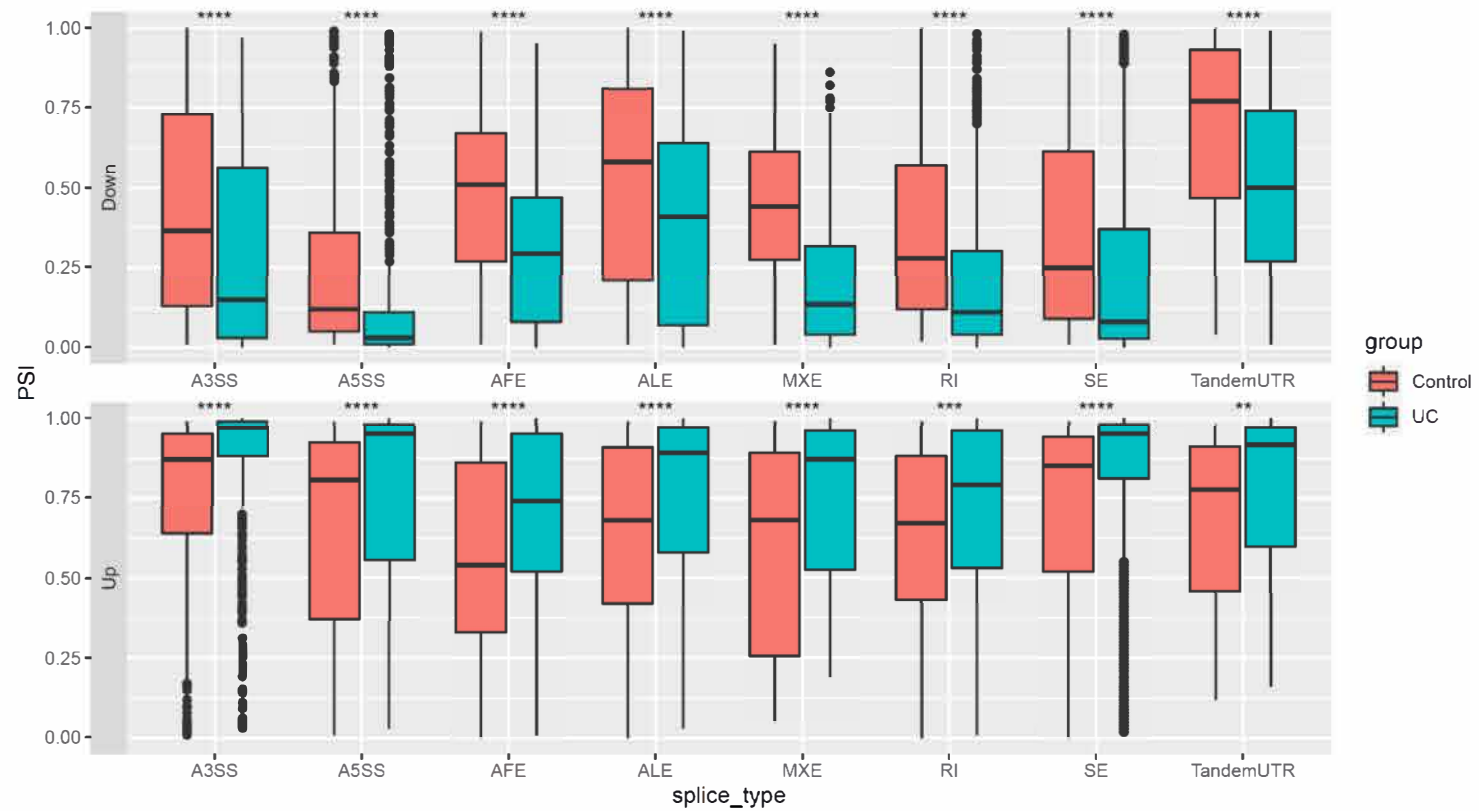

S3

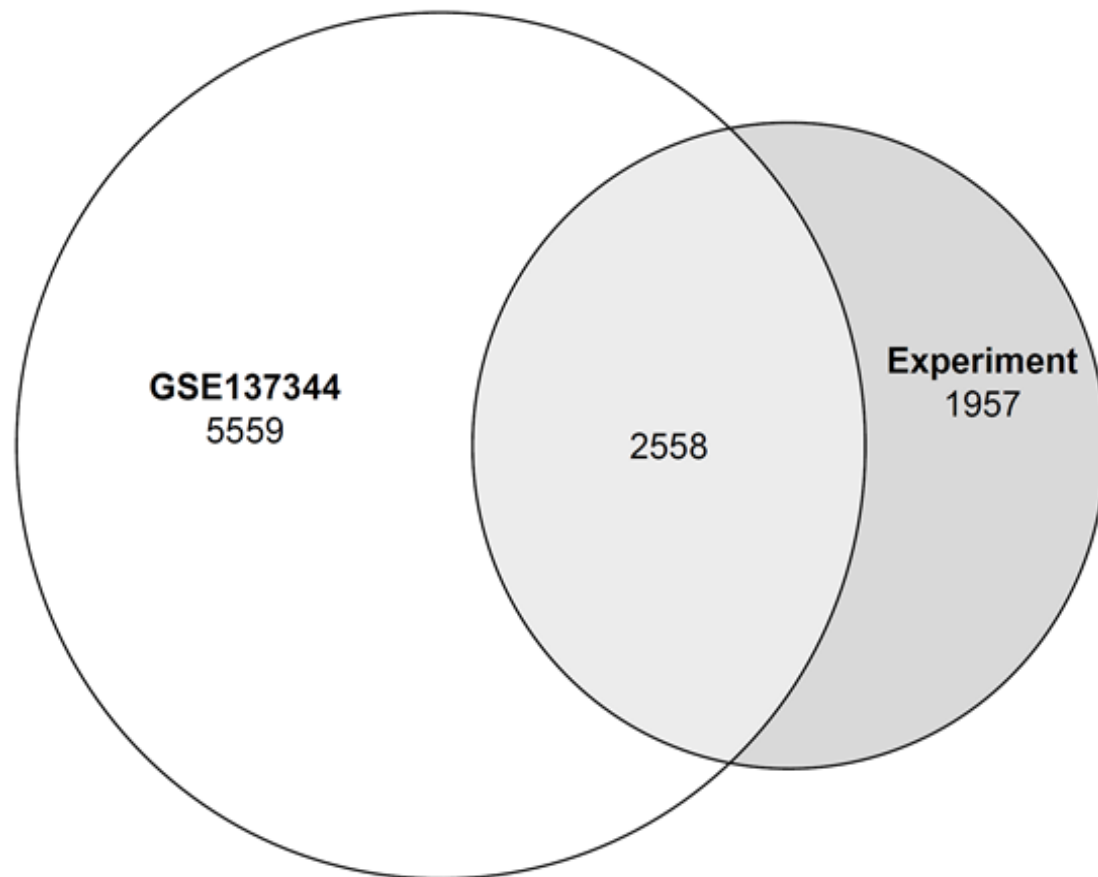

### UC-vs-Ctrl

Bioconductor package EnhancedVolcano

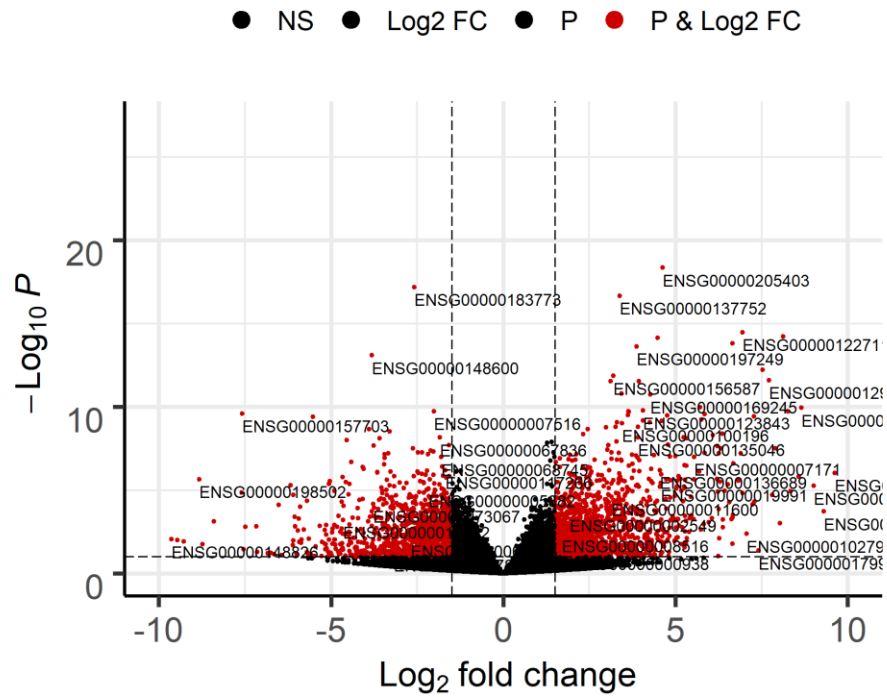

Total = 39815 variables

S5

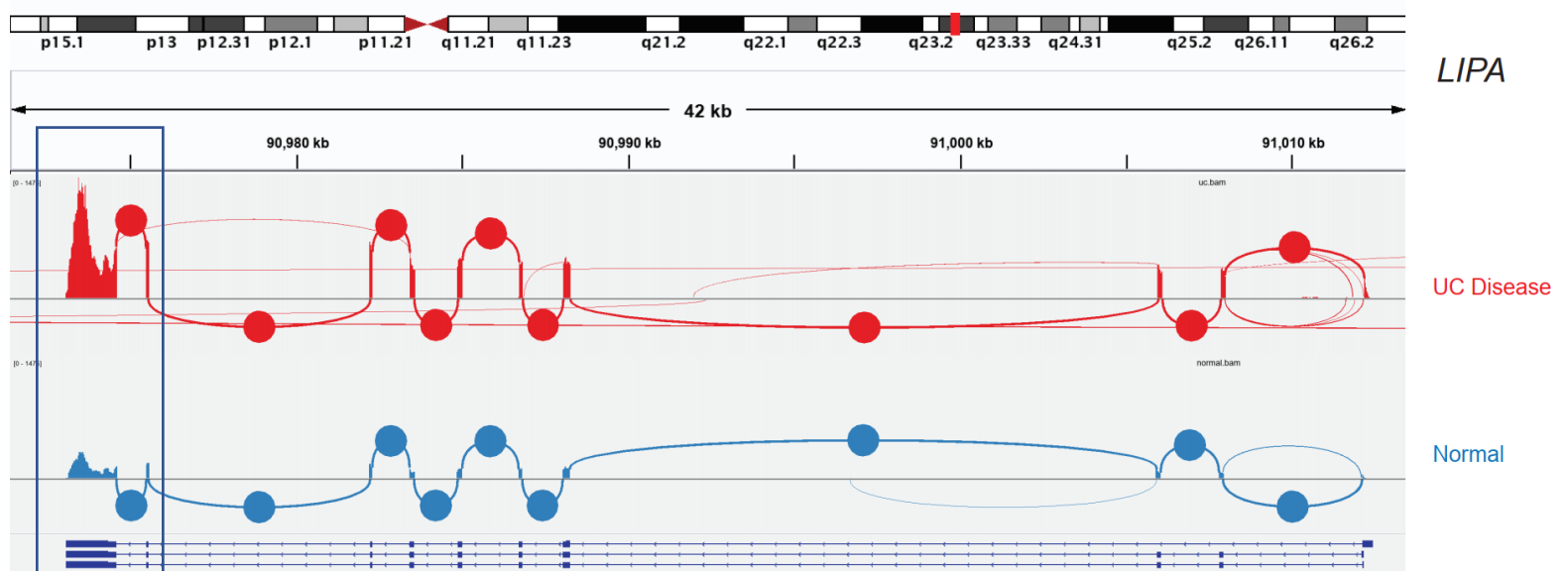

Supplement: Supplementary file 1 — Additional file 1: Figure S1. PSI comparison of significant AS event between UC and control samples in the GSE137344 public dataset. Figure S2. PSI comparison of significant AS event between UC and control samples in the 8-sample mRNA-seq experiment. Figure S3. Genes with significant AS event that overlap with the public dataset. Figure S4. Volcano plot showed more than 1,654 genes were significantly regulated in UC patients compared to the control. Figure S5. Sashimi plot presented tandem 3’UTR events in LIPA gene in our validation experiments. [file 12938_2021_959_MOESM1_ESM.pdf]
