# Supplementary material for: Dysregulation of alternative splicing is associated with the pathogenesis of ulcerative colitis
Source: Biomed Eng Online. 2021 Nov 27;20:121. doi: 10.1186/s12938-021-00959-4 (PMC8627048; doi:10.1186/s12938-021-00959-4)
Supplement: Supplementary file 5 — Additional file 5: Table S4. Raw counts of 4 significantly dysregulated splicing factors expression data from the public dataset and the validation experiment RNA-seq (UC versus Control); FC = Foldchange. The splicing events on mRNA are mainly regulated by splicing factors. We collected 404 splicing factors from (PMID: 29617667). [file 12938_2021_959_MOESM5_ESM.docx]

Table S4. Raw counts of 4 significantly dysregulated splicing factors expression data from the public dataset and the validation experiment RNA-seq (UC versus Control); FC = Foldchange

| Gene symbol | Description | FC in GSE137344 study | FC in Validation experiment |
| --- | --- | --- | --- |
| ELAVL3 | ELAV Like RNA Binding Protein 3 | 2.693878 | 1.529412 |
| ZMAT5 | Zinc Finger Matrin-Type 5 | 1.466667 | 1.402985 |
| BAG2 | BAG Cochaperone 2 | 1.430424 | 2.78515 |
| HSPA5 | Heat Shock Protein Family A (Hsp70) Member 5 | 1.408052 | 3.89345 |
| TXNL4A | Thioredoxin Like 4A | 1.392116 | 1.51447 |

The splicing events on mRNA are mainly regulated by splicing factors. We collected 404 splicing factors from (PMID: 29617667).
